# Genome-Wide Association Study of Tan Spot Resistance in a Hexaploid Wheat Collection From Kazakhstan

**DOI:** 10.3389/fgene.2020.581214

**Published:** 2021-01-11

**Authors:** Alma Kokhmetova, Deepmala Sehgal, Shaukat Ali, Makpal Atishova, Madina Kumarbayeva, Irina Leonova, Susanne Dreisigacker

**Affiliations:** ^1^Laboratory of Breeding and Genetics, Institute of Plant Biology and Biotechnology (IPBB), Almaty, Kazakhstan; ^2^Faculty of Agronomy, Kazakh National Agrarian University, Almaty, Kazakhstan; ^3^Global Wheat Program, International Maize and Wheat Improvement Center (CIMMYT), Texcoco, Mexico; ^4^Department of Agronomy, Horticulture & Plant Science, South Dakota State University, Brookings, SD, United States; ^5^Institute of Cytology and Genetics of the Siberian Branch of the Russian Academy of Sciences, Novosibirsk, Russia

**Keywords:** DArTseq, genome-wide association study, *Pyrenophora tritici-repentis*, tan spot, wheat

## Abstract

Tan spot, caused by *Pyrenophora tritici-repentis*, is a serious foliar disease of wheat in Kazakhstan with reported yield losses as high as 50% during epidemic years. Here, we report the evaluation of a collection of 191 hexaploid spring and winter wheat lines for tan spot resistance and its underlying genetic architecture using genome-wide association study (GWAS). Our wheat collection comprised candidate varieties from Kazakhstan, Russia, and CIMMYT. It was genotyped using the DArTseq technology and phenotyped for resistance to tan spot at seedling and adult plant stages in Kazakhstan. DArTseq SNPs revealed high genetic diversity (average polymorphic information content = 0.33) in the panel and genome-wide linkage disequilibrium decay at 22 Mb (threshold *r^2^* = 0.1). Principal component analysis revealed a clear separation of Eurasian germplasm from CIMMYT and IWWIP lines. GWAS identified 34 marker-trait associations (MTA) for resistance to tan spot and the amount of phenotypic variation explained by these MTA ranged from 4% to 13.7%. Our results suggest the existence of novel valuable resistant alleles on chromosomes 3BS, and 5DL and 6AL for resistance to Race 1 and Race 5, respectively, in addition to known genes *tsn1* and *tsc2.* On chromosome 6AL, a genomic region spanning 3 Mb was identified conferring resistance to both Race 1 and Race 5. Epistatic interaction of associated loci was revealed on chromosomes 1B, 5B, 7B, 5A, and 6A contributing to additional variation of 3.2–11.7%. Twenty-five lines with the best allele combinations of SNPs associated with resistance to both races have been identified as candidates for future variety release and breeding. The results of the present study will be further validated in other independent genetic backgrounds to be able to use markers in breeding.

## Introduction

Bread wheat (*Triticum aestivum* L.) is grown in more than 85 countries with a gross annual production of 761.5 million tons (FAO, 2020). Consumed by more than 40% world population, it is the primary source of calories for millions of people worldwide. Central Asia, including Kazakhstan, plays a significant role in regional and global food security as most of the grain produced is traded in these regions (Morgounov et al., 2014). The total area sown to wheat in Kazakhstan represents over 85% of total cereal production. Tan spot caused by *Pyrenophora tritici-repentis* (Died.) is an economically important disease in most wheat-growing regions worldwide, including Europe, North and South America, Australia, and Asia (Duveiller et al., 2005). In Central Asia, the disease was discovered in the 1980s in Tajikistan (Khasanov, 1988), and since then, it has spread throughout Central Asia and Kazakhstan (Postnikova and Khasanov, 1998; Lamari et al., 2005; Koyshibaev, 2018). On an average, losses due to tan spot vary from 10% to 15% but may reach up to 50% during epidemic years (Rees et al., 1982; Shabeer and Bockus, 1988). Tan spot reduces total yield, grain weight, number of grains per head, total biomass, and grain quality (Shabeer and Bockus, 1988; Fernandez et al., 1994).

Currently, eight races of *P. tritici-repentis* (PTR) have been identified based on necrosis and chlorosis symptoms induced by host-selective toxins (HST) on a set of differential wheat varieties (Lamari et al., 2003). Races 2, 3, and 5 can be designated as basic races, while Races 1, 6, 7, and 8 are combinations of the three basic races except for Race 4, which is avirulent (Lamari et al., 2003). To date, three host-specific toxins, Ptr ToxA, Ptr ToxB, and Ptr ToxC, of *P. tritici-repentis* have been identified and well characterized. Ptr ToxA is produced by Races 1, 2, 7, and 8 (Lamari et al., 2003) and associated with necrotic symptoms in Ptr ToxA-sensitive cultivars. Ptr ToxB is produced by Races 5, 6, 7, and 8 and is responsible for the induction of chlorosis in Ptr Tox-B-sensitive cultivars. Both Ptr ToxA and Ptr ToxB are proteins in nature, while Ptr ToxC (produced by Race 1 and Race 3) is a non-ionic, polar non-protein (Effertz et al., 2002).

There are a number of studies conducted on the racial composition of *P. tritici-repentis* worldwide (Lamari and Bernier, 1989; Ali and Francl, 2002, 2003; Lamari et al., 2003, 2005; Maraite et al., 2006; Gamba et al., 2012; Kokhmetova et al., 2016, 2017). The greatest diversity in the pathogen population was observed in Azerbaijan, where Races 1, 2, 3, 5, 7, and 8 were identified, and in Syria, where Races 1, 3, 5, 7, and 8 were observed. Race 1 is the most widespread race in Central Asia and Kazakhstan (87%), and Races 2, 3, and 4 are prevalent infrequently (Maraite et al., 2006). Recently, Race 8 was also found in high frequency in Kazakhstan (Kokhmetova et al., 2016, 2017).

The inheritance of resistance to tan spot is both qualitative and quantitative; toxicity resistance genes and quantitative trait loci (QTL) are known (Faris et al., 1996, 2013; Gamba and Lamari, 1998; Anderson et al., 1999; Faris and Friesen, 2005; Tadesse et al., 2006a,b; Chu et al., 2008, 2010; Singh et al., 2008, 2010, 2016; Li et al., 2011; Hu et al., 2019). Qualitative genes identified through conidial inoculations have been given the designation “*Tsr*” for “tan spot resistance,” and genes associated with reaction to HST-containing cultures are designated as “*Tsc*” or “*Tsn*” depending on the necrosis or chlorosis symptom exhibited by the HST (McIntosh et al., 2008). To date, eight major *Tsr* genes (*Tsrl*, *Tsr2*, *Tsr3*, *Tsr4*, *Tsr5*, *Tsr6*, *TsrHar*, and *TsrAri*) located on chromosomes 2BS, 3AS, 3BL, 3DS, and 5BL have been identified (McIntosh et al., 2013). Two *Tsc* genes (*Tsc1 and Tsc2*), conferring sensitivity to Ptr ToxC and Ptr ToxB, have been mapped on chromosomes 1AS (Effertz et al., 2002) and 2BS (Friesen and Faris, 2004; Abeysekara et al., 2010), respectively.

With the availability of millions of single-nucleotide polymorphisms (SNPs) in almost all crops, genome-wide association study (GWAS) has become a common approach in dissecting genetic architecture of traits and in identifying beneficial alleles for use in marker-assisted selection (Santure and Garant, 2018). In wheat, GWAS has been conducted for several diseases, including resistance to *Stagonospora nodorum blotch* (Tommasini et al., 2007), stem rust (Bajgain et al., 2016; Elbasyoni et al., 2017), stripe rust (Yu et al., 2011, 2012; Sehgal et al., 2016), fusarium head blight (Miedaner et al., 2011), and *Septoria tritici blotch* (Gerard et al., 2017). GWAS studies for tan spot resistance have led to significant advances in the identification of loci on chromosomes 2B, 3B, 4A, 6B, 6A, and 7B (Gurung et al., 2011; Patel et al., 2013; Kollers et al., 2014; Singh et al., 2016; Hu et al., 2019). However, hitherto, the genetic basis of tan spot resistance in wheat germplasm from Kazakhstan has not been yet investigated. Our objectives were therefore (1) to evaluate a Kazakhstan collection of winter and spring wheat cultivars/breeding lines for tan spot resistance, (2) to identify genetic loci associated with tan spot resistance via GWAS, (3) to explore gene-by-gene interactions among significant genetic loci, and (4) to identify best combinations of alleles and lines for future phenotyping trials and breeding.

## Materials and Methods

### Plant Materials

A total of 191 spring and winter hexaploid wheat accessions (*Triticum aestivum* L.) were evaluated in this study. The collection included 111 cultivars and breeding lines from Kazakhstan, 17 cultivars from Russia, 1 cultivar from Brazil, 30 lines released by CIMMYT, and 31 lines released by CIMMYT-ICARDA-IWWIP (International Maize and Wheat Improvement Center-International Center for Agricultural Research in the Dry Areas–International Winter Wheat Improvement Program) (Supplementary Table 1). The collection comprised important wheat genotypes that have been widely used as parental lines in breeding programs across the Kazakhstan and Central Asian countries. The 111 cultivars from Kazakhstan included in the collection were chosen based on their contrasting phenotypic expression for traits of agronomic and disease resistance. Three differential lines, cultivar “Glenlea” carrying the *Tsc1* gene, line “6B662” carrying *Tsc2*, and cultivar Salamouni resistant to all known races and insensitive to toxins Ptr ToxA, Ptr ToxB, and Ptr ToxC were included as checks.

### Fungal Isolates, Inoculum Production, and Inoculations

The isolates Pti2 and DW7 used in this study were previously obtained from bread wheat (*T. aestivum* L.) and durum wheat (*Triticum durum* Desf), respectively, and stored as dried mycelial plugs at −20°C (Jordahl and Francl, 1992). The isolate Pti2 was from spring wheat and the isolate DW7 was collected from a durum wheat field in North Dakota (Ali and Francl, 2003), and they were used for inoculum production in this study. The inoculum and inoculations were carried out as described in Ali and Francl (2001). To prepare the inoculum, a single mycelial plug (0.5 cm in diameter) was placed on V8PDA (150 ml of V8 juice, 10 g of Difco PDA, 10 g of Difco agar, 3 g of calcium carbonate, and 850 ml of sterile distilled water) (Lamari and Bernier, 1989) in 9-cm petri plates. After placing the mycelia plug of both isolates individually in petri plates, the plates were wrapped with aluminum foil and incubated at 21°C for 5 days. Thereafter, the petri plates were filled with sterilized distilled water, and hyphal growth was knocked down with a flamed-sterilized glass test tube. After suppressing the hyphae, excess water was removed from the plates, and then they were incubated in an alternate cycle of 24 h light at 22°C and 24 h dark at 16°C to induce conidiophores and conidia. The conidia were dislodged with an inoculating loop wired needle. The conidial suspension was obtained by adding 30 ml of distilled sterile water in each plate, and conidia were dislodged with a looped wire needle. The conidial suspension was adjusted to 3,000 spores/ml using the hemocytometer. One drop of Tween 20 was added/100 ml of conidial suspension before inoculation. Inoculated seedlings were moved to a mist chamber at 21°C with a 16-h photoperiod for 24 h to enhance the chances of infection. The chamber was misted for 16 s at 3-min intervals to keep 100% RH. The plants then were moved to a growth chamber at 22°C.

### Seedling Test

Seedlings of all 191 wheat accessions were raised in containers (3.8 cm in diameter and 20 cm long) as described in Ali and Francl (2001). For each race of *P. tritici-repentis* (Races 1 and 5), separate experiments were conducted in the growth chamber. Three seeds were planted in a plastic container (Stuewe & Sons, Inc., OR) filled with Sunshine Mix #1 (Fison Horticulture, Vancouver, BC, Canada). All containers were placed in 96-slot racks to hold them straight. Each container was considered as an experimental unit, and each single plant in a container with three seedlings served as an entry. All entries were arranged in a randomized complete design with three replications. Thus, nine seedlings of each genotype were evaluated individually at the two-leaf stage against Race 1 (isolate Pti2) and Race 5 (isolate DW7). In each experiment, replications were treated as random effect and the wheat accessions as fixed effects. All experiments were conducted in growth chambers at South Dakota State University (SDSU), Brookings, SD, in 2015. Seedlings were rated 8 days post-inoculation using the rating scale 1–5 (Lamari and Bernier, 1989) where 1–2 is resistant to moderately resistant, 3 is moderately susceptible, and 4–5 is susceptible. BLUE (best linear unbiased estimators) across replications were calculated in META-R (Vargas et al., 2013).

### Phenotyping for Sensitivity to Ptr ToxA and Ptr ToxB

The 191 wheat entries and three checks were tested for their reaction to purified toxins Ptr ToxA and Ptr ToxB at a concentration of 10 μg/ml. Ptr ToxA and Ptr ToxB were kindly provided by Dr. Steven Meinhardt, North Dakota State University, Fargo, and Dr. Timothy Friesen, USDA, Fargo, ND. Four leaves (second leaf fully expanded), of each genotype were infiltrated as described by Faris et al. (1996) with pure Ptr ToxA and Ptr ToxB culture filtrates separately. After infiltration, the plants were kept in a growth chamber at 21°C during the day and 18°C at night with 16 h photoperiod in the growth chamber. Leaves evaluated 4 days post infiltration and scored as insensitive (−) or sensitive (+). This experiment was repeated twice. Wheat genotypes “Glenlea” (ToxA sensitive), “6B662” (ToxB sensitive), and “Salamouni” (ToxA and ToxB insensitive) were also included in the experiment to verify the results and toxin viability (Ali and Francl, 2003; Lamari et al., 2003).

### Field Phenotyping

The experimental material was phenotyped at the Kazakh Research Institute of Agriculture and Plant Growing (KRIAPG), Almalybak (43°13′09′′ N, 76°36′17′′ E) in Southeast Kazakhstan, Almaty region, during the 2016 to 2018 cropping seasons. Experiments were conducted as a completely randomized design with three replicates. Individual plot size was 1 m^2^. Fertilizer treatments, 60 and 30 kg/ha of N and P_2_O_5_, respectively, and other management practices were corresponded to those normally recommended for the region (Dospekhov, 1985).

Experiments were planted in mid-September and were harvested in mid-August the following year for 3 years. The weather conditions at Almalybak were characterized annually by over 400 mm of rainfall. The irrigated foothill zone, where KRIAPG is located, is a relatively high moistured location; the experimental wheat materials were irrigated thrice during the growing season and were kept free from weeds.

The field evaluation was carried out under natural epidemic conditions in 2016 and 2017, whereas in 2018, it was carried out under natural conditions as well as using artificial inoculation. Ten Flag-1 leaves were evaluated for each disease assessment of genotypes. The disease was assessed three times. The experiments on an artificial infectious background were made with naturally infected straw stubbles. In October, before sowing, the infected straw (1 kg/m^2^) was incorporated into the soil. For evaluation of field response, disease severities were assessed on first leaves and flag leaves in GS 65–69, Zadoks scale (Zadoks et al., 1974). The percentage of Ptr-infected leaf area was determined on each leaf, and the average value for all evaluated leaves was calculated for each wheat entry in order to determine the PTR score. A rating system based on% leaf area infected developed for appraising the foliar intensity of diseases (Kremneva and Volkova, 2007) was used to categorize host reaction to *P. tritici-repentis*. This scale of disease severity was rated numerically based on% necrotic or chlorotic area as follows: 0–10%—resistant (R), 11–20%—moderately resistant (MR), 21–30%—moderately susceptible (MS), and 31–100%—susceptible (S). The standard wheat differentials included “Glenlea” (susceptible check) and “Salamouni” (resistant check) and were included in the field trials. Plant height (PH) was recorded in centimeters from the soil surface to the tip of the spike of 10–15 plants per plot in GS 90–99. Days to heading (DH) were recorded as the number of days from planting to the 50% spike emergence in GS 49–50 (Zadoks et al., 1974).

Analysis of phenotypic resistance ratings to tan spot based on the area under disease progress curve (AUDPC) was performed. AUDPC was calculated annually by summarizing the progress of disease severity. AUDPC values from double digit and AUDPC from flag leaf (F) and penultimate leaf (F-1) were separately calculated by using the following formula described by Das et al. (1992). ANOVA analyses of phenotypic data was done using linear mixed effect model with package *lme4* in R using replications as fixed effect and entries as random effect. The package *reshape* was used to transform the data for ANOVA analysis. Broad-sense-heritability was estimated using the formula: *h*^2^ = *V*_g_/(*V*_g_ + *V*_err_/*r*), where *V*_g_ is the genotypic variance, *V*_err_ is the error variance, and r = the number of replications.

### DNA Extraction and Genotyping

Genomic DNA was extracted from fresh leaves collected from a single individual plant using a modified CTAB (cetyltrimethylammonium bromide) method described in the CIMMYT laboratory protocols (Dreisigacker et al., 2012, 2016) and quantified using a NanoDrop 8000 spectrophotometer V 2.1.0. Genotyping was performed using the DArTseq^TM^ technology provided by the Genetic Analysis and Service for Agriculture (SAGA) laboratory in Mexico. Briefly, the genotypes were sequenced at 192-plexing on Illumina HiSeq2500 with 1 × 77-bp reads. A proprietary analytical pipeline developed by DArT P/L was used to generate allele calls for SNP and presence/absence variation (PAV) markers (Sansaloni et al., 2011). A 100K consensus map provided by SAGA was used to obtain genetic positions of the SNPs (Sansaloni et al. unpublished). To obtain physical positions of SNPs, sequence reads of the SNPs were blasted to the reference genome of RefSeq V1.0 in the Ensemble Plants database^1^.

### Diversity, Population Structure, and Linkage Disequilibrium Analysis

Polymorphic information content (PIC) (Botstein et al., 1980) was calculated to characterize the genetic diversity of the panel using PowerMarker version 3.25 (Liu and Muse, 2005). Population structure was investigated by principal component analysis (PCA) using the STATS package in R and using STRUCTURE v 2.3.4 (Pritchard et al., 2000). The STRUCTURE program was run by setting replication number to 50,000 for the burn-in and the Markov Chain Monte Carlo (MCMC) periods. *K* values were run from 1 to 7 using “admixture” and “correlated allele frequency” models. The correct estimation of *K* was provided by an *ad hoc* statistic delta *K*, calculated using the program STRUCTURE HARVESTER^2^. A weighted neighbor joining tree was constructed in DARwin version 6.0^3^.

GAPIT v. 2.0 (Lipka et al., 2012) was used to obtain squared correlation coefficient (*r*^2^), a measure of linkage disequilibrium (LD), for all pairwise comparisons among markers. Pattern of LD decay was visualized in individual subpopulations and in the whole panel by plotting pair-wise *r*^2^ values against the physical distance. A smooth line was fit to the data using second-degree locally weighted scatterplot smoothing, LOESS (Breseghello and Sorrells, 2006) as implemented in SAS. For the LOESS estimation of LD decay, genetic distance was estimated as the point where the LOESS curve first crosses the baseline *r*^2^ of 0.1.

### Genome-Wide Association Analysis

The BLUE and average scores for disease severity at seedling stage and AUDPC values at adult plant stage in the field were used for conducting GWAS. To map Ptr ToxA and Ptr ToxB resistance loci, all insensitive genotypes were scored as 1 and all sensitive genotypes as 5, and the average of the two scores was used for GWAS. VanRaden algorithm (VanRaden, 2008) was used to calculate the kinship matrix in the GAPIT package vs. 2.0 (Lipka et al., 2012). GWAS was conducted in the TASSEL software vs. 5 (Bradbury et al., 2007). A mixed linear model (MLM) was applied in which PCA was a fixed variate (first three PCs) and kinship as random. A false-discovery rate (FDR) was used to assess the significance of the p value (<0.05). The allelic effect of significant marker-trait associations was estimated as the difference between the mean value of lines with and without favorable allele and was presented as box plots. A second GWAS analysis was conducted in which we included 8,947 unmapped markers along with a filtered set of markers. Interestingly, eight unmapped markers crossed the FDR threshold, but these showed very low R^2^ values, ranging from 2.2 to 3.1%. We have not shown these results.

### Epistatic Interaction Analysis

Two- and three-locus epistatic interactions among associated SNPs and among genome-wide SNPs were explored using a custom-made in-house script described in Sehgal et al. (2017). Briefly, a stepwise multiple regression was performed using a linear regression model to calculate *P* values for pairwise marker interactions. A threshold of *P* < 0.0001 was used to declare significant marker–marker interactions. The parameter *R*^2^ was used to describe percentage variation explained by the significant interactions. The interactions showing percentage variation <10% were discarded from the output.

## Results

### Resistance to *Pyrenophora tritici-repentis* Race 1 and Race 5 at Seedling Stage

Uniform and consistent tan spot development was observed in the seedling evaluation in growth chambers. ANOVA showed significant differences among genotypes (*P* < 0.001) for the reaction scores to both races (Supplementary Table 2). The checks Glenlea and 6B662 developed necrosis and chlorosis to Race 1 and Race 5, respectively, and verified the Race identity and the success of inoculation. Salamouni did not develop any symptoms and exhibited resistant reaction. Salamouni and Glenlea showed scores of 1.0 and 4.5 for Race 1, whereas for Race 5, Salamouni and 6B-662 showed scores of 1.1 and 4.3.

**FIGURE 1:**
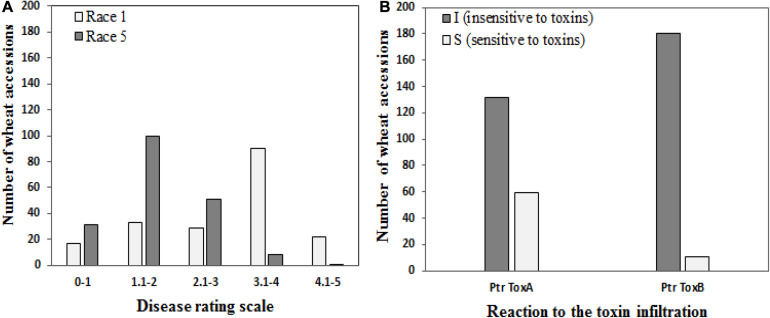
Frequencies of 191 wheat genotypes in different disease score groups **(A)** to tan spot (*Pyrenophora tritici-repentis* Race 1 and Race 5) and **(B)** host-selective Ptr ToxA and Ptr ToxB.

Of the 191 genotypes, 50 genotypes (26.2%) revealed a disease score of less than 2 and were considered resistant to Race 1, whereas 130 wheat accessions (68.6%) were resistant to Race 5 (Figure 1A). Sixty-two accessions showed resistance against both races.

### Sensitivity to Ptr ToxA and Ptr ToxB

One hundred thirty-two genotypes (69.1%) showed no symptoms of necrosis and were determined to be Ptr ToxA insensitive, while the remaining 59 genotypes (30.9%) were scored as sensitive (Figure 1B). Salamouni and Glenlea exhibited insensitive and sensitive reactions to Ptr ToxA, respectively. Ptr ToxA sensitivity was associated with disease susceptibility in field conditions in all years (Pearson’s correlation *r* = 0.22 to 0.38, *P* < 0.001).

For Ptr ToxB, Salamouni and 6B-662 exhibited insensitive and sensitive reactions, respectively. Twelve genotypes (6.3%) exhibited chlorosis in the toxin-infiltrated leaf area and were rated to be Ptr ToxB sensitive. The remaining 93.7% (179) did not develop chlorosis symptoms and were rated as Ptr ToxB insensitive (Figure 1B). The correlation of Ptr Tox B sensitivity with disease susceptibility in the field was not significant (*r* = 0.03–0.11, *P* = 0.3029).

### Field Resistance to *Pyrenophora tritici-repentis*

**FIGURE 2:**
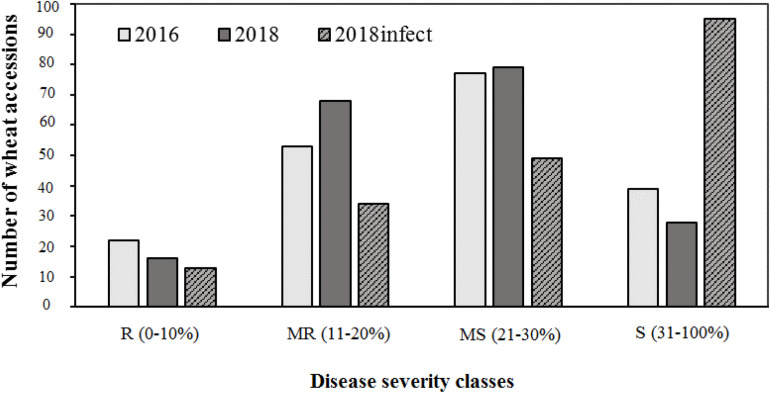
Frequencies of 191 wheat genotypes reaction to tan spot in field.

Of the three field experiments conducted in 2016, 2017, and 2018 under natural epidemic conditions, the disease pressure was minimum in 2017, and more than 95% of lines were resistant. Hence, phenotypic data from 2017 was not used for any further analysis, i.e., calculating AUDPC scores or GWAS. There were significant (*P* < 0.001) differences for tan spot response in the field in 2016 and 2018 (under both natural conditions and under artificial inoculation in 2018). Days to heading (DH) and plant height (PH) also showed significant variation (*P* < 0.001) (Supplementary Table 3). Field severity of tan spot varied between three experiments (2016–2018). The disease severity was generally higher under artificial inoculation conditions (2018_infect) than under natural inoculum conditions in 2016 and 2018. The number of accessions in each reaction class across three experiments is presented in Figure 2. On an average, 9, 27, 44, and 25% of genotypes were resistant (R), moderately resistant (MR), moderately susceptible (MS) and susceptible (S) in the field. Under artificial inoculation conditions in 2018 (2018_infect), the number of accessions in class S increased to 73%. Six genotypes, i.e., Tungysh, JAC161/TEMU51.80, TOO11/TOOOO7, SOMO/SORA/ACTS5, NANJING 82149/KAUZ, and ALTAR 84/AE. SQUARROSA exhibited the highest level of resistance. A significant number of wheat entries from CIMMYT (13%) demonstrated high or moderate level of resistance. Correlation of days to heading and plant height with AUDPC scores was not significant in all field seasons (Supplementary Table 4).

Comparison of the phenotypic data under field conditions with the green house experiment showed that out of 69 entries that showed R and MR response in field, 31 and 62 genotypes had disease scores less than 2 for Race 1 and Race 5, respectively.

### Marker Distribution and Diversity

A total of 40,429 SNPs were obtained across the 191 genotypes after allele calling. Of these, 10,186 markers with missing data >20% and 8,947 unmapped markers were removed from the dataset. Further, markers with minor allele frequency (MAF) <0.05 and >0.95 were culled. Three lines, CATBIRD, BR35/BR14, and KR12-5001, showing more than 25% missing data were also culled, and a final filtered set of 8,154 SNPs on 187 lines was utilized for further analyses. Marker distribution on the 21 wheat chromosomes, PIC, and LD statistics are shown in Table 1. The least number of markers were distributed on the D genome (11.2%). The highest numbers of markers were found on chromosome 2B (870; 10.7%), followed by chromosome 5B (769; 9.4%) and 3B (762; 8.1%) (Table 1).

**TABLE 1:** Polymorphic information content (PIC) and linkage disequilibrium (LD) estimated for all chromosomes using 8,154 GBS markers.

**Chromosome**	**Number of SNPs**	**Average PIC**	**Total number of marker pairs with *r^2^* ≥ 0.1**	**Average LD at *r^2^* ≥ 0.1**
1A	464	0.34	4,521	0.31
1B	608	0.36	6,249	0.47
1D	167	0.31	2,488	0.48
2A	579	0.30	4,312	0.38
2B	870	0.36	8,016	0.34
2D	295	0.29	6,322	0.56
3A	469	0.37	3,911	0.32
3B	662	0.36	6,548	0.29
3D	129	0.31	828	0.45
4A	367	0.36	5,108	0.39
4B	202	0.35	1,747	0.38
4D	28	0.33	45	0.35
5A	442	0.33	5,498	0.40
5B	769	0.36	6,631	0.36
5D	79	0.31	621	0.36
6A	412	0.35	3,542	0.33
6B	476	0.38	5,567	0.30
6D	109	0.30	733	0.37
7A	565	0.34	5,924	0.34
7B	354	0.37	3,173	0.30
7D	108	0.28	495	0.41

The average PIC for the 8,154 SNPs was 0.33. Comparison of the average PIC of winter versus spring wheat lines showed that diversity was lower in winter wheat (0.38) compared to spring wheat germplasm (0.41).

### Population Structure and Linkage Disequilibrium Decay

**FIGURE 3:**
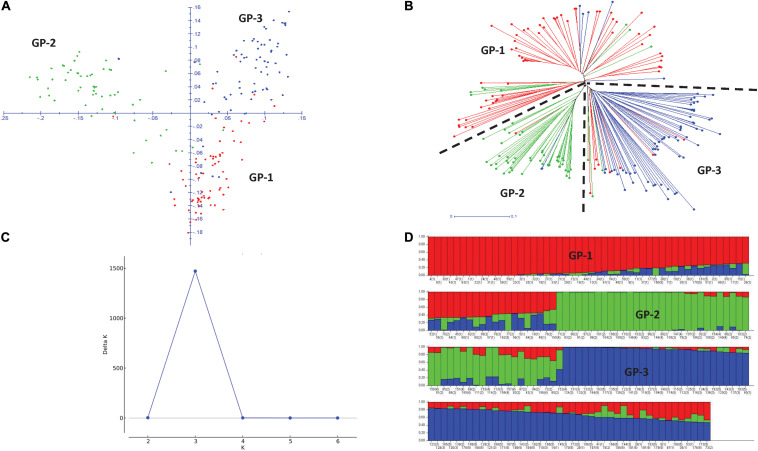
Two-dimensional principal component analysis plot **(A)** and weighted neighboring-joining dendrogram **(B)** of panel showing three groups with both analyses. GP1, Winter type from Kazakhstan; GP2, Spring type from Kazakhstan and Russia; GP3, Spring and winter types from the CIMMYT and CIMMYT-ICARDA-IWWIP program. Delta *K* vs. *K* plot showing the best *K* at 3 **(C)** and bar plot showing subpopulation division at *K* = 3 **(D)**.

Principal component analysis (PCA) revealed three broad groups in the panel, and the first three principal components explained 11.8, 9.9, and 5.5% of the genetic variation, respectively (Figure 3A). Groups 1 and 2 containing Eurasian germplasm with winter and spring wheat lines, respectively, were separated from the group of lines developed by the CIMMYT and CIMMYT-ICARDA-IWWIP program (Group 3). The weighted neighbor-joining (NJ) dendrogram confirmed the three groups obtained in PCA (Figure 3B). Further Δ*K* vs. *K* plot, based on results obtained in Bayesian model-based STRUCTURE analysis, showed the highest likelihood at *K* = 3 (Figure 3C). At *K* = 3, the three subpopulations corresponded with both PCA and NJ-based groups (Figure 3D).

The average LD was highest in the D genome (0.42) and was higher compared to A (0.35) and B (0.34) genomes. Genome-wide LD decay patterns were investigated in the three subpopulations individually and in the whole panel (Supplementary Figure 1). The results revealed that LD decay was faster in the CIMMYT and IWWIP germplasm, i.e., Group 3 (18 Mb at cut off *r*^2^ = 0.1) compared to Eurasian lines (30 and 25 Mb in groups 1 and 2, respectively). The LD decayed at 22 Mb in the whole panel (Supplementary Figure 1D).

### Marker-Trait Associations

#### MTA for Resistance to Race 1 and Race 5 at Seedling Stage

Ten SNPs on chromosomes 1B (3), 3B (1), 4B (1), 5A (1), 5B (1), 6A (1), and 7B (2) were associated with resistance to Race 1 of *P. tritici-repentis* with 7.3–10.5% variation explained (Table 2 and Supplementary Figures 2A,B). The phenotypic mean difference between the alleles for the significant SNPs ranged from 0.2 to 1.3. The SNP identified on chromosome 6A with clone ID 1004240 showed the strongest allele effect followed by SNPs on chromosomes 4B (ID 3958510) and 3B (ID 1147153). Favorable alleles at these loci were present in 5–9% frequencies.

**TABLE 2:** Markers associated with resistance to Race 1, Race 5, and insensitivities to Ptr ToxA and Ptr ToxB at seedling stage.

**Trait**	**Marker name (clone ID)**	**Chr**	**Genetic position on consensus map (cM)**	**Physical position (Chinese_ Spring_v1.0)**	***P*-value**	***R*^2^**	**Allele 1**	**Mean**	**Allele 2**	**Mean**	**Fav. allele frequency**
Race 1 (Average)	1103424	1BS	86.7	62,284,931	3.29E–04	7.3	T	3.0	C	2.0	0.13
	1406319	1BS	90.8	–	3.18E–04	7.7	C	3.0	T	2.2	0.17
	1090475	1BS	92.2	57,766,497	6.18E–04	7.9	G	3.0	C	2.0	0.13
	1147153	3BS*	53.7	227,835,794	9.28E–04	8.6	C	3.1	G	2.2	0.05
	3958510	4BS	39.4	–	8.61E–04	9.2	T	3.0	C	1.9	0.09
	3946488	5BL	40.8	–	9.37E–04	8.5	T	3.6	C	2.8	0.80
Race 1 (BLUE)	1134493	5AL	153.01	698,528,378	4.61E–04	8.1	T	3.0	C	2.7	0.51
	1004240	6AL	89.8	602,914,149	5.60E–04	10.5	T	3.1	C	1.8	0.08
	3021234	7BL	49.4	–	1.17E–04	8.2	T	3.1	C	2.5	0.28
	1081730	7BL	49.4	538,125,772	4.53E–04	8.3	G	3.1	A	2.5	0.32
Race 5 (Average)	1008802	5AL	44.1	441,476,773	3.09E–04	6.8	G	2.0	C	1.7	0.23
	1093048	5DL*	151.1	629,968,953	8.65E–04	7.7	T	1.9	G	1.4	0.10
	1862737	6AL*	90.3	599,831,232	6.50E–04	8.0	G	2.4	C	1.7	0.79
Race 5 (BLUE)	1008802	5AL	44.1	441,476,773	7.60E–04	7.2	G	2.1	C	1.7	0.23
Insensitivity to Ptr ToxA (Average)	4003201	2BS	86.6	–	7.34E–05	7.0	T	2.8 (sensitive)	C	2.2 (insensitive)	0.60
	1138872	5BL	35.7	429,865,271	9.06E–06	10.7	G	2.4 (sensitive)	T	1.4 (insensitive)	0.11
	3955588	5BL	70.1	546,831,358	6.98E–06	13.5	G	3.7 (sensitive)	T	1.3 (insensitive)	0.59
	1128605	5BL	73.1	547,522,421	3.20E–05	13.7	G	3.3 (sensitive)	T	1.6 (insensitive)	0.46
Insensitivity to Ptr ToxB (average)	1038630	2AS	18.09	29,263,722	1.42E–05	9.1	C	2.3 (sensitive)	G	1.2 (insensitive)	0.86
	1095982	2BS	75.08	–	8.52E–05	10.6	A	2.6 (sensitive)	G	1.1 (insensitive)	0.87
	1065699	2BS	71.8	184,907,691	3.57E–04	9.4	C	2.4 (sensitive)	T	1.1 (insensitive)	0.77
	1093535	3AS*	41.1	64,777,406	7.95E–04	8.6	T	2.0 (sensitive)	C	1.2 (insensitive)	0.81
	2263392	3BL	156.6	824,374,808	7.51E–04	8.0	C	2.0 (sensitive)	T	1.2 (insensitive)	0.83
	1217569	4AL	29.8	–	4.14E–05	7.2	C	1.8 (sensitive)	T	1.2 (insensitive)	0.90

*Asterisk represent novel quantitative trait loci (QTL) identified in the study, – BLAST identified either a different chromosome than reported based on consensus map provided by Genetic Analysis and Service for Agriculture (SAGA) or reported single nucleotide polymorphism (SNP) was not identified in the Ensemble Plants database.*

For Race 5, three genomic regions on chromosomes 5A, 5D, and 6A were identified to be associated with percentage variation from 6.8 to 8.0% (Table 2 and Supplementary Figures 2C,D). Compared to associations identified for Race 1, markers associated with Race 5 showed lower phenotypic mean differences between alleles. Interestingly, the genomic region spanning 3 Mb on chromosome 6AL was identified to be associated with resistances to both races, Race 1 and Race 5. This indicates the importance of chromosome 6AL in imparting race-specific resistance to *P. tritici-repentis*. *In silico* analysis of the genomic region identified on chromosome 6A revealed two candidate gene hits, TraesCS6A02G37880 and TraesCS6A02G384600 with SNPs 1004240 and 1862737, respectively, both with oxidoreductase activity. Oxidoreductase family proteins are known as important components of pathogen-associated molecular pattern-triggered immunity *via* production of reactive oxygen species in response to pathogen attack (Heiser and Elstner, 2002). Of the two candidate genes, TraesCS6A02G384600 has been investigated for expression analysis (Expression Atlas^4^), and it is known to express in outer pericarp layer of developing grain.

**FIGURE 4:**
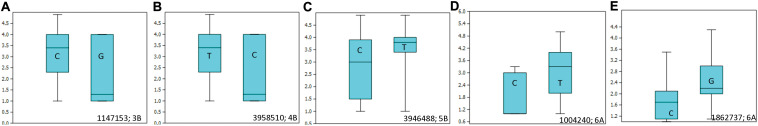
Box plots showing allelic effects of important associations identified for resistance to Race 1 with average **(A–C)** and best linear unbiased estimator (BLUE) scores **(D)** and for resistance to Race 5 with average scores **(E)**. The number on the right side below shows the name of the associated marker and chromosome number.

Allelic effects of important associations identified for resistance to Races 1 and 5 are shown in Figure 4. Twenty-five tan spot resistant lines have been identified with different allelic combinations for seedling resistance to Race 1 and Race 5 (Supplementary Table 5).

#### Marker-Trait Associations for Insensitivity to Toxins Ptr ToxA and Ptr ToxB

Four MTA were identified associated with insensitivity to Ptr ToxA on chromosomes 2B (1) and 5B (3) with percentage variation explained by them ranging from 7.0 to 13.7% (Table 2). The phenotypic mean difference between the alleles for the three significant SNPs on chromosome 5B ranged from 1.0 to 2.4, whereas for the SNP on chromosome 2B, the allelic difference in alleles was 0.6. For insensitivity to Ptr ToxB, important loci were identified on chromosomes 2A (1), 2B (2), 3A (1), 3B (1), and 4B (1) with percentage variation from 7.2 to 10.6%. The QTL identified on chromosome 2B related to SNPs 1095982 and 1065699 had the strongest allelic effect among all (Table 2).

#### Marker-Trait Associations for Area Under Disease Progress Curve Scores From Three Field Experiments

Analysis of AUDPC for two seasons (2016 and 2018) under natural epidemic conditions revealed eight associations on chromosomes 1B (1), 2B (4), 3B (1), 4A (1), and 6B (1) (Table 3). On chromosome 2B, four MTA were identified, which were delineated into two QTL, one between 18.5 and 28.0 cM associated with 2018 AUDPC scores and the second between 86.5 and 89.8 cM associated with 2016 AUDPC scores. The second QTL located 86.5–89.8 cM explained higher (8.7–11.9%) percentage variation compared to the other 2BS QTL (4.0–7.3%). In addition, the association identified on 1B also explained high percentage variation (11.6%). Under artificial inoculation conditions in 2018, two associations were identified on chromosomes 3A (1) and 5B (1), both explaining moderate percentage variation. QQ plots for all traits investigated by GWAS are shown in Supplementary Figure 3.

**TABLE 3:** Markers associated with area under disease progress curve (AUDPC) scores under natural epidemic conditions in two field seasons (2016 and 2018) and by artificial inoculation in 2018 (AUDPC 2018_infect).

**Trait**	**Marker name (clone ID)**	**Chr**	**Genetic position on consensus map (cM)**	**Physical position (Chinese_Spring_ v1.0)**	***P*-Value**	***R*^2^**	**Allele 1**	**Mean**	**Allele 2**	**Mean**	**Fav. allele frequency**
AUDPC 2016	1022665	2BS	86.5	760,015,871	6.94E–05	11.9	T	23.1	C	16.6	0.75
	1367534	2BS	89.8	758,922,449	8.47E–04	8.7	G	20.7	A	16.6	0.72
	2256531	4AL	103.3	–	8.74E–04	7.2	C	19.4	A	16.4	0.61
AUDPC 2018	4993225	1BS	87.3	57,643,782	2.51E–05	11.6	G	19.3	C	7.7	0.12
	3533672	2BS	18.5	–	1.27E–04	7.3	C	18.3	T	11.3	0.11
	1061674	2BS	28.0	33,729,208	2.47E–04	4.0	G	17.8	C	14.0	0.05
	2295410	3BL	145.1	–	5.64E–04	6.0	G	18.3	A	14.2	0.58
	3020636	6BS	6.0	–	6.65E–05	9.7	C	18.9	A	11.2	0.23
AUDPC 2018_infect	4988948	3AL*	130.9	731,226,020	9.19E–04	7.2	T	59.0	C	44.0	0.27
	1234099	5BL	60.1	531,195,246	3.01E–04	9.9	G	55.2	T	37.2	0.13

*Asterisk represent novel QTL identified for adult plant resistance in the study, – BLAST identified either a different chromosome than reported based on consensus map provided by SAGA or reported SNP was not identified in Ensemble Plants database.*

### Epistatic Interactions

**FIGURE 5:**
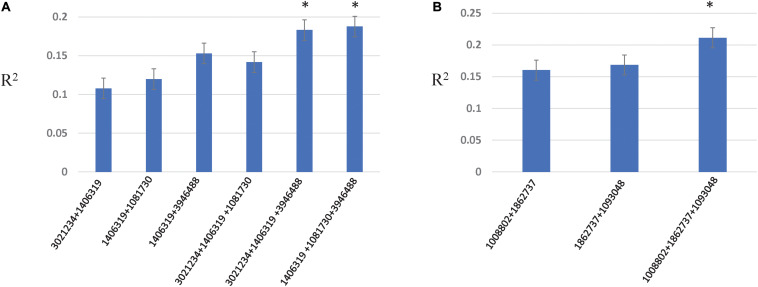
Additive epistatic interactions among associated loci for resistance to Race 1 **(A)** and Race 5 **(B)**. The *X*-axis represents two- and three-marker combinations interacting epistatically. The *Y*-axis represents the percentage variation as *R*^2^ explained by marker combinations. The asterisk represents the marker combinations that resulted in the highest *R*^2^.

Two- and three-locus interactions were estimated among associated loci, and among associated and genome wide loci (Figure 5, Supplementary Table 6, and Supplementary Figures 4–6). For Race 1 and Race 5, associated loci showed epistatic interactions among them with *R*^2^ explained from 10.7 to 18.7% for Race 1 and 16.0–21.1% for Race 5, respectively (Figure 5). The *R*^2^ explained by marker combinations was higher than individual markers for both Race 1 and Race 5. Further, the three-marker combination resulted in the highest additive interactions explained by *R*^2^ of 18.7 and 21.1% for Race 1 and Race 5, respectively. For other traits, no significant interactions were observed among associated loci. However, significant interactions among unassociated genome-wide markers were observed for all traits except insensitivity to Ptr ToxB (Supplementary Figures 4–6).

## Discussion

Tan spot, caused by *P. tritici-repentis*, is a serious foliar disease affecting wheat production in Kazakhstan, especially in the northern region of the country where farmers lose anywhere from 10% to as much as 50% of their crop due to this disease (Koyshibaev, 2018). It has been predicted that outbreaks of this disease in Kazakhstan are likely to increase in severity and frequency due to an increase in mean annual temperatures and altered precipitation patterns (Salnikov et al., 2015). Characterization of the current germplasm for resistance to prominent races of the pathogen in Kazakhstan has therefore become more imperative than ever before (Kokhmetova et al., 2018, 2019). Here, we evaluated registered cultivars and lines from Kazakhstan and Russia representing promising spring and winter wheat germplasm along with lines released by the CIMMYT and CIMMYT-ICARDA-IWWIP program.

Most wheat entries evaluated in the study showed a susceptible response to Race 1 (72.6%), whereas for Race 5, 67.5% of lines were resistant. It is a common observation in most germplasm collections (Liu et al., 2015; Halder et al., 2019), and our results reinforce that finding resistance against Ptr Race 1 is very challenging compared to other races. Race 1 is the most prevalent worldwide (Ali and Francl, 2003; Abdullah et al., 2017) and is reported to contain the virulence of Races 2 and 3, making it more aggressive than other races (Lamari et al., 2003). Nevertheless, 25 lines (13%) resistant to both races (Race 1 and Race 5) have been identified in the present study. Regarding toxin sensitivities of the genotypes, a low but positive correlation was observed between Ptr ToxA sensitivity of the genotypes and disease susceptibility in the field, which is in contrast with the observations made by Friesen et al. (2003); Faris and Friesen (2005), Noriel et al. (2011), and Halder et al. (2019). We did not obtain any significant correlations between days to heading and plant height with Ptr resistance in field, suggesting that none of these phenological traits significantly affected tan spot resistance in the germplasm investigated. Li et al. (2011) and Pandey et al. (2018) obtained similar results, i.e., no association of tan spot resistance with phenological traits. Many previous studies have detected a negative correlation between heading date or plant height with disease resistance (Srinivasachary et al., 2009; Mao et al., 2010; Kollers et al., 2013a,b), including tan spot resistance (Kollers et al., 2014). The broad sense heritability (*h*2) estimates for tan spot across years and different infectious background were high (from 0.83 to 0.91) indicating that resistance to tan spot can be improved by selection (Supplementary Table 3). Similar heritability estimates for tan spot disease reaction have been reported by Singh et al. (2016, 2019). Heritability ranged from 0.70 to 0.96 for DH and was 0.98 for PH in different years. Jamil et al. (2019) reported heritability estimates for these traits in a similar range.

The mean PIC of the panel based on 8,154 SNPs was 0.33, which is in the same range as observed recently in wheat cultivars from China (Gao et al., 2016) and higher than those obtained in United States wheat cultivars (Chao et al., 2009, 2010) or other germplasm sets (Somers et al., 2007; Alemu et al., 2020). The higher diversity can be explained by the diverse origin of the lines included in the panel such as those from CIMMYT. CIMMYT germplasm has shown substantial genetic diversity in previous studies (Dreisigacker et al., 2005; Reif et al., 2005; Warburton et al., 2006; Sehgal et al., 2015). Population structure through PCA, NJ, and STRUCTURE analyses of the panel revealed a clear distinction of spring and winter wheat types from Kazakhstan. Chao et al. (2010) also obtained distinct groups of spring and winter wheat from the US. A third group of spring and winter wheat was exclusive to lines released by the CIMMYT and IWWIP program, thus clearly separating CIMMYT germplasm from Eurasian lines. The panel, therefore, shows a moderate population structure along with high diversity rendering it fit for trait dissection using the GWAS approach.

The pattern of LD across the three genomes revealed the highest LD in the D genome followed by the A and B genomes. This pattern is common in wheat and reflects the recombination history of the three genomes and population bottleneck accompanying the origin of hexaploid wheat (Akhunov et al., 2010; Chao et al., 2010). Genome-wide LD decay was observed at 22 Mb in the whole panel, which is in the same range as reported recently in a CIMMYT spring wheat collection (Jamil et al., 2019) and in a composite collection made of CIMMYT and South Asian genotypes (Phuke et al., 2020). It was not possible to compare the LD decay of the panel with other germplasms because LD decay has been reported as a measure of genetic distance (in cM) in previous studies (Somers et al., 2007; Benson et al., 2012; Chen et al., 2012; Würschum et al., 2013; Edae et al., 2014; Zegeye et al., 2014; Sehgal et al., 2017; Erginbas-Orakci et al., 2018). A comparison of LD decay among three subpopulations of the present study revealed a faster decay in the subpopulation composed by CIMMYT and IWWIP lines compared to the two subpopulations of Eurasian lines, which is attributed to higher diversity of CIMMYT and IWWIP germplasm vis-à-vis Eurasian germplasm. Breeders at CIMMYT have successfully broadened the genetic diversity of the elite germplasm through incorporation of primary synthetics into the breeding programs and consistent introductions of additional materials from all over the world (Sehgal et al., 2015). Similarly, the pedigree of IWWIP lines incorporates diverse CIMMYT parents and a wide range of genetically un-related winter wheat from Turkey and Iran, Russia, Ukraine, Romania, Bulgaria, Hungary, and United States winter wheat.

Genome-wide association study identified a total of 34 MTA for tan spot resistance. Of all the MTA identified for seedling resistance to Races 1 and 5, the genomic region on chromosome 6AL showed the biggest phenotypic effect. In fact, two MTA were identified within a 3-Mb genomic region on 6AL for resistance to both Race 1 (Clone ID 1004240) and 5 (Clone ID 1862737) (Table 2). The favorable allele of the SNP with Clone ID 1004240 was predominantly present in CIMMYT lines and three Kazakhstani winter wheat types (Naz/GF55-5, 428 g/MK-122A-2 and Almaly/Obri), while the favorable allele of the SNP with Clone ID 1862737 was predominant in Kazakhstani winter and spring wheat lines. Singh et al. (2016) reported two QTL on 6AL (6AL_1_ and 6AL_2_) for tan spot resistance in CIMMYT germplasm for resistance to Race 1, and the SNP with clone ID 1004240 represents 6AL_2_ QTL. The allelic effect obtained by this SNP is, however, three times larger than reported by Singh et al. (2016). The second marker on chromosome 6AL (Clone ID 1862737) represents a novel QTL for resistance to Race 5 because the only QTL reported on 6A for resistance to Race 5 (Gurung et al., 2014) is on 6AS. For resistance to Race 1, MTA on chromosomes 4BS and 3BS also showed strong effects. Halder et al. (2019) recently reported a novel QTL on 4BS for resistance to Race 1 in the United Kingdom Watkins core collection, and the associated marker identified here on chromosome 4BS corresponds to this QTL. No gene or QTL has so far been reported on chromosome 3BS for resistance to Race 1. Hence, MTA obtained on chromosome 3BS is likely to be novel.

The collection investigated was comprised of 12% spring wheat lines distributed by CIMMYT and evaluated to be resistant to both *Septoria tritici blotch* and tan spot. In CIMMYT’s germplasm, a large QTL on chromosome 1BS (∼19 cM based on consensus map and ∼130 Mb based on physical position) has contributed significantly for resistance to Race 1, which was suggested due to the 1B.1R translocation harboring several resistance genes including *Lr26*, *Yr9*, *Sr31*, *Pm8*, and others (Singh et al., 2016; Juliana et al., 2018). The three MTA identified here on chromosome 1BS covered ∼5 Mb on reference genome and were in the middle of 1BS QTL. Similarly, MTA identified on other chromosomes for resistance to Race 1 and Race 5 coincided with known QTL except the one on chromosome 5DL for resistance to Race 5 (Faris et al., 2013; Singh et al., 2016; Hu et al., 2019). The only reported QTL on chromosome 5DL is for resistance to Race 1 (Faris et al., 2012). Therefore, the associated marker identified on chromosome 5DL for resistance to Race 5 represents a new QTL.

Isolates of Race 1 and Race 5 are known to produce toxins Ptr ToxA and Ptr ToxB, respectively. Qualitative resistance in the host has been reported to be manifested by two toxin insensitivity genes on chromosomes 5BL (*tsn1*) and 2BS (*tsc2*), conferring resistance against Ptr ToxA and Ptr ToxB, respectively (Singh et al., 2010; Faris et al., 2013). We obtained three MTA on chromosome 5BL associated with insensitivity to Ptr Tox A, which were within the same genomic region as the *tsn1* gene (Faris et al., 2010). Of the three, two SNPs (Clone ID 3955588 and 1128605) had higher allelic effects on toxin insensitivity compared to the third SNP (Clone ID 1138872). An additional locus for insensitivity to Ptr ToxA was identified on chromosome 2BS, which coincided with previous QTL reported for resistance to Race 1 (Gurung et al., 2014). For insensitivity to Ptr ToxB, important associations were obtained on chromosomes 2AS, 2BS, 3AS, 3BL, and 4AL. Of all associations, two SNPs (clone IDs 1095982 and 1065699) on 2BS showed the strongest allelic effect and were in the same genomic region as the cataloged *tsc2* gene. The SNP on chromosome 2AS was the second most important genomic region with a strong allelic effect on insensitivity to Ptr ToxB. Previous studies till date have reported only minor effect QTLs on 2AS for insensitivity to Ptr Tox B and/or resistance to Race 5 (Friesen and Faris, 2004; Chu et al., 2008). On chromosome 3AS, *tsr4* has been mapped, and the associated marker obtained here on chromosome 3AS is around 70 cM from *tsr4* locus and hence unlikely to be in the location of *tsr4* gene. The known genes on chromosome 3BL are *tsn2* and *tsn5* that confer resistance to the necrosis induced by Race 3 and Race 5, respectively (Singh et al., 2008). The locus obtained on chromosome 3BL is of moderate effect and corresponds to minor QTL reported by Faris and Friesen (2005) and Chu et al. (2010) on the same chromosome. The locus identified on chromosome 4AL also represents another minor effect QTL as also previously reported for resistance to Race 5 (Friesen and Faris, 2004).

Regarding MTA identified for AUDPC scores, most associations except the one on chromosome 3AL were identified within 2–3 Mb genomic region of the markers associated with seedling resistance to Races 1 and 5 or insensitivities to toxins Ptr ToxA and Ptr ToxB in the present study, thus confirming to be major genes (*tsn 1*, *tsc2*). Three associations on chromosomes 2BS, 4AL, and 6BS overlap with minor QTL reported for tan spot resistance in previous studies (Gurung et al., 2011; Halder et al., 2019). The QTL found on chromosome 3AL is likely new for adult plant resistance, which was identified only at adult plant stage in the present study.

The role of epistasis in the genetic architecture of disease resistance has been investigated for stem rust resistance and stripe rust resistance in wheat using both biparental and GWAS designs (Kolmer et al., 2011; Yu et al., 2011). However, investigations on the contribution of epistatic interactions to the genetic architecture of tan spot resistance have been rarely investigated (Singh et al., 2019). Singh et al. (2019) revealed epistatic interactions between QTL on chromosomes 1B and 5B and between QTL on chromosomes 1A and 6A for seedling resistance to Race 1 in a biparental population, however, with small effects. We confirm significant epistatic interactions between associated loci on chromosomes 1B and 5B in the present study with even larger percentage variation explained (up to 18.7%). In addition, we obtained significantly higher interactions between associated loci on chromosomes 1B and 7B with a percent variation explained as large as 14.1% for resistance to Race 1. The associated locus on 7B (SNP 1081730) was also involved in genome-wide interactions with other loci on chromosomes 7A, 2D, and 3B, and contributed to additional 4% variation. For seedling resistance to Race 5, associated loci on chromosomes 5A, 5D, and 6A contributed to additive epistatic interactions of 21.1%. These results indicated that both additive and epistatic effects are important for tan spot resistance to both races, Race 1 and Race 5. The results also suggest that three marker combinations identified for resistance to Race 1 and Race 5 can be used efficiently in marker-assisted selection.

## Conclusion

Our results suggest the existence of valuable resistant alleles on chromosomes 3AS, 3AL, 3BS, and 6AL for tan spot resistance in the investigated germplasm in addition to known genes *tsn1* and *tsc2.* The study therefore confirms that resistance to tan spot in the collection is attributed to both the known toxin insensitivity genes with major effects as well as broad-spectrum and race-non-specific genes, which have minor effects. Twenty-five tan spot-resistant lines have been identified with different allelic combinations for resistance to Race 1 and Race 5 for future variety release. The candidate genes identified on 6A are important targets for future validation studies for tan spot resistance.

## Data Availability Statement

The genotyping data is available at the CIMMYT Research Data and Software Repository Network: https://hdl.handle.net/11529/10548482.

## Author Contributions

AK conceived the manuscript and designed the research. DS and AK analyzed the data and wrote the manuscript. SA, IL, MA, and MK generated the phenotypic data. DS and SD generated the allele called GBS marker data. All authors reviewed the manuscript.

## Conflict of Interest

The authors declare that the research was conducted in the absence of any commercial or financial relationships that could be construed as a potential conflict of interest.

## Footnotes

1 https://plants.ensembl.org/Triticum_aestivum/Info/Index

2 http://taylor0.biology.ucla.edu/structureHarvester/

3 https://darwin.cirad.fr/product.php

4 https://www.ebi.ac.uk/gxa/home

## Supplementary Material

Supplementary Figure 1 Linkage disequilibrium decay plot for the three subpopulations; CIMMYT and IWWIP lines **(A)**, Spring wheat from Kazakhstan and Russia **(B)**, Winter wheat from Kazakhstan **(C)** and across all panel **(D)**. *X*-axis represents physical distance in Mb; *Y*-axis represents correlation coefficient.

Supplementary Figure 2 Manhattan plots showing significant markers associated with BLUE (left) and average (right) scores for resistance to Race 1 **(A,B)** and Race 5 **(C,D)**, respectively.

Supplementary Figure 3 QQ plots of all traits used in GWAS analysis with mixed linear model by using first three principal components as a fixed variate and kinship as a random variate.

Supplementary Figure 4 Genome wide epistatic interactions for resistance to Race 1 **(A)**, Race 5 **(B)**, and sensitivity to Ptr Tox A **(C)**. For sensitivity to Ptr ToxB, no significant interactions were observed.

Supplementary Figure 5 Genome wide epistatic interactions for AUDPC scores in field seasons in 2016 **(A)** and 2018 **(B)**.

Supplementary Figure 6 Genome wide epistatic interactions for AUDPC 2018_infect; 1 associated SNP on 3A showed genome wide and explained 5.5%.

Supplementary Table 1 Cultivars and breeding lines used in the present study.

Supplementary Table 2 Analysis of variance for tan spot (TS) disease scores for resistance to Race 1 and Race 5 and distribution of tan spot score in checks and the panel.

Supplementary Table 3 Analysis of variance (ANOVA) for tan spot severity and phenological traits under natural conditions and under artificial inoculation conditions and summary statistics.

Supplementary Table 4 Correlations of days to heading (DH) and plant height (PH) with AUDPC scores in field seasons.

Supplementary Table 5 Kazakhstani and Russian entries with resistances to Race 1 and Race 5 with best allele combinations.

Supplementary Table 6 Epistatic interactions among associated loci for tan spot resistance.
