# Supplementary figures and images for: Genome-Wide Association Study of Tan Spot Resistance in a Hexaploid Wheat Collection From Kazakhstan
Source: Front Genet. 2021 Jan 11;11:581214. doi: 10.3389/fgene.2020.581214 (PMC7831376; doi:10.3389/fgene.2020.581214)

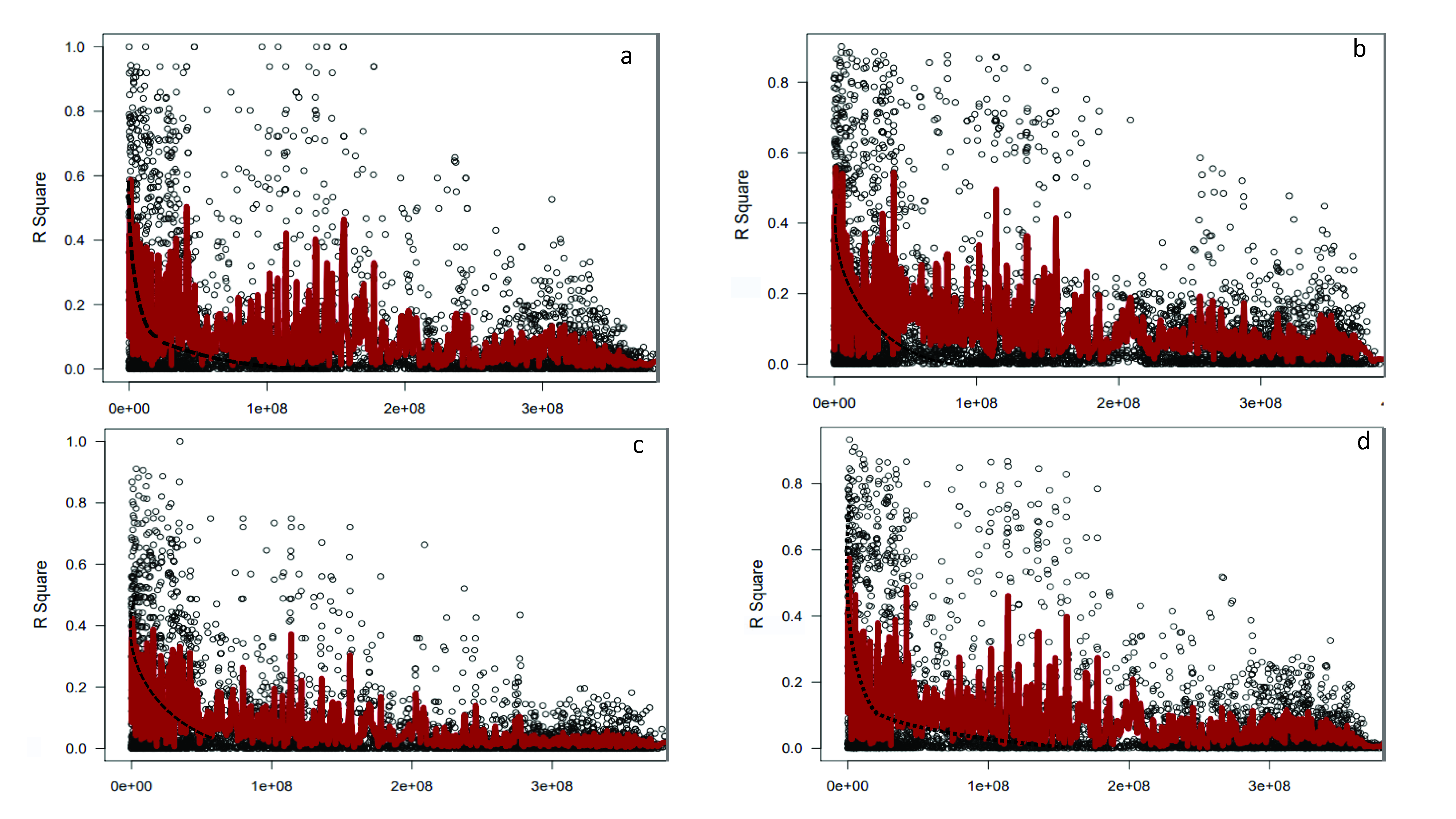

Supplement: Supplementary Figure 1 — Linkage disequilibrium decay plot for the three subpopulations; CIMMYT and IWWIP lines (A), Spring wheat from Kazakhstan and Russia (B), Winter wheat from Kazakhstan (C) and across all panel (D). X-axis represents physical distance in Mb; Y-axis represents correlation coefficient. [file Image_1.TIFF]

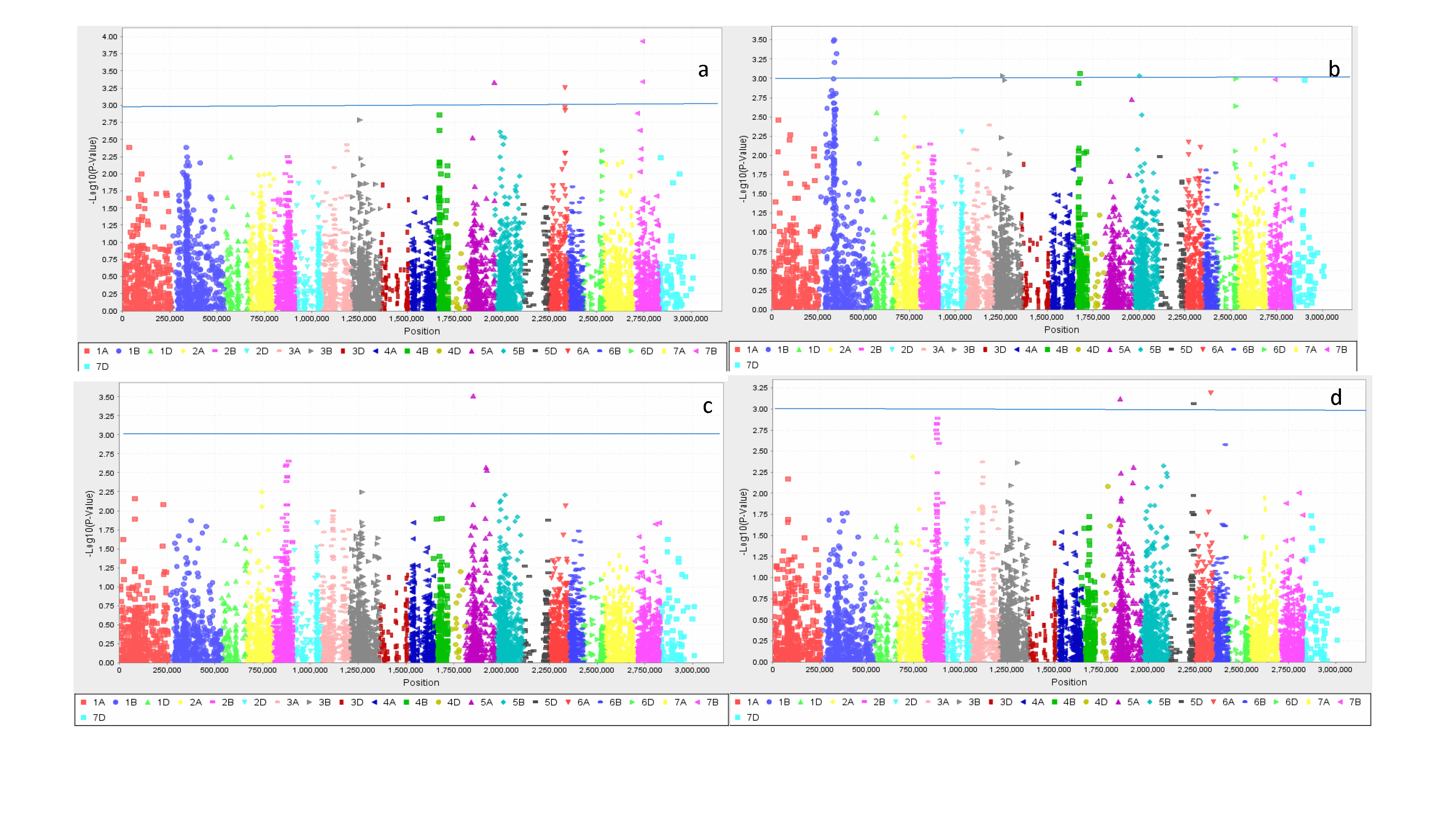

Supplement: Supplementary Figure 2 — Manhattan plots showing significant markers associated with BLUE (left) and average (right) scores for resistance to Race 1 (A,B) and Race 5 (C,D), respectively. [file Image_2.TIFF]

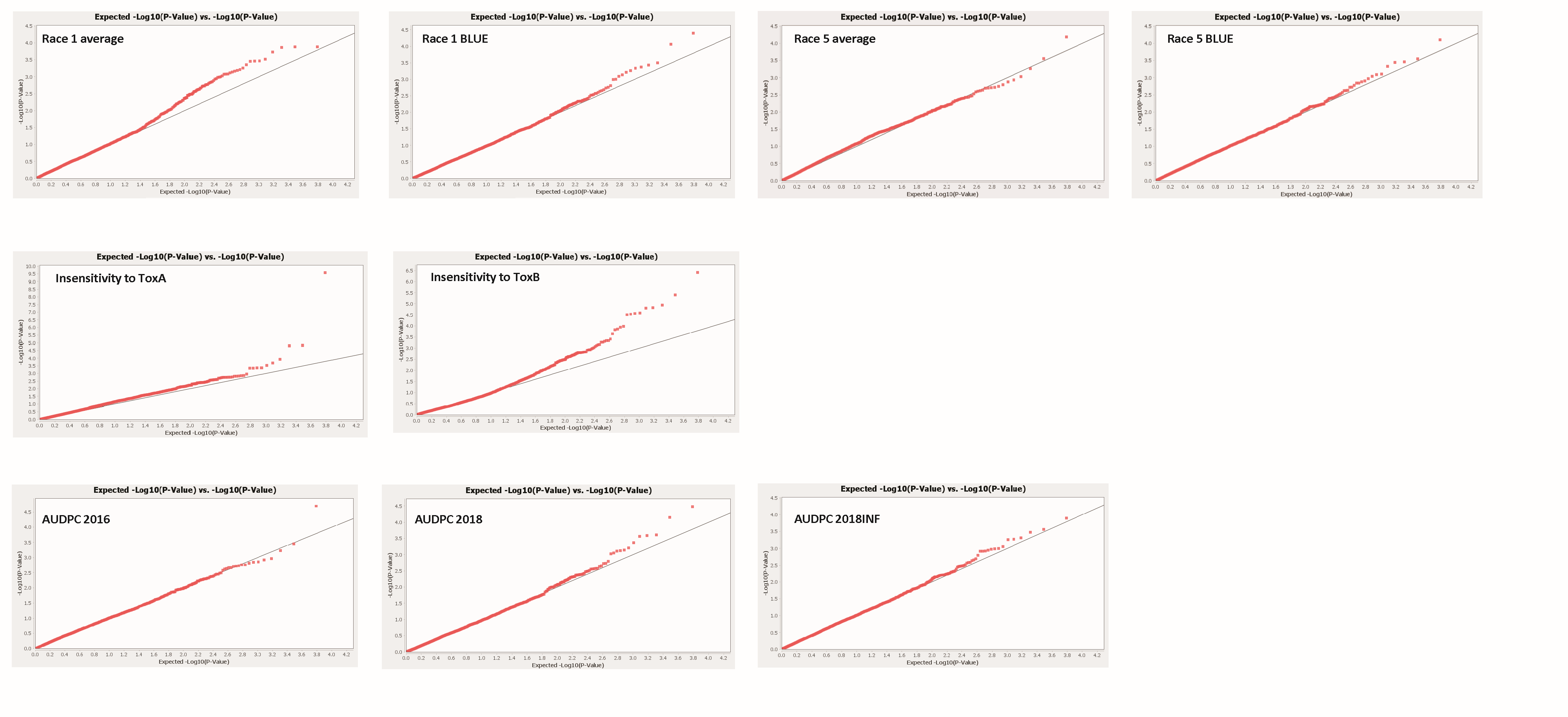

Supplement: Supplementary Figure 3 — QQ plots of all traits used in GWAS analysis with mixed linear model by using first three principal components as a fixed variate and kinship as a random variate. [file Image_3.TIFF]

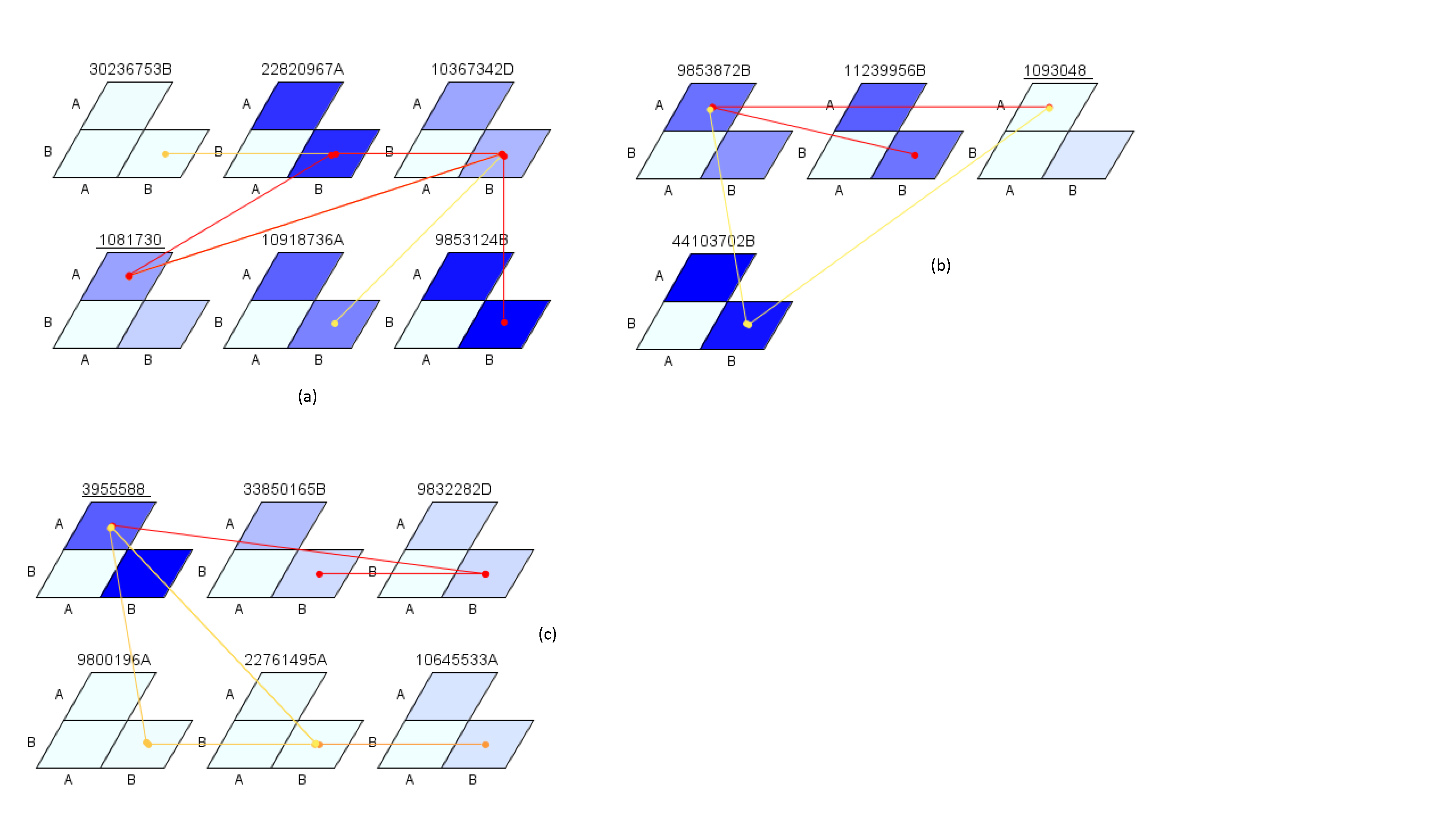

Supplement: Supplementary Figure 4 — Genome wide epistatic interactions for resistance to Race 1 (A), Race 5 (B), and sensitivity to Ptr Tox A (C). For sensitivity to Ptr ToxB, no significant interactions were observed. [file Image_4.TIFF]

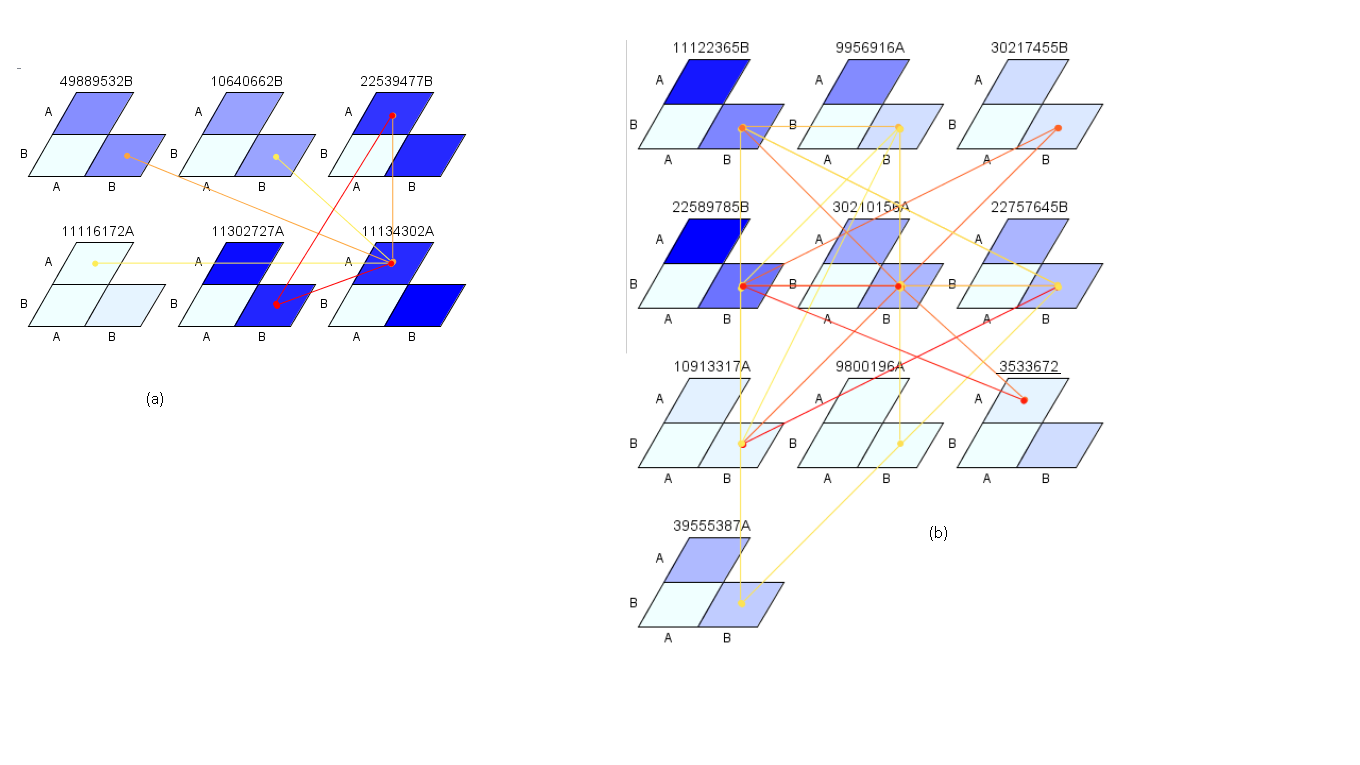

Supplement: Supplementary Figure 5 — Genome wide epistatic interactions for AUDPC scores in field seasons in 2016 (A) and 2018 (B). [file Image_5.TIFF]

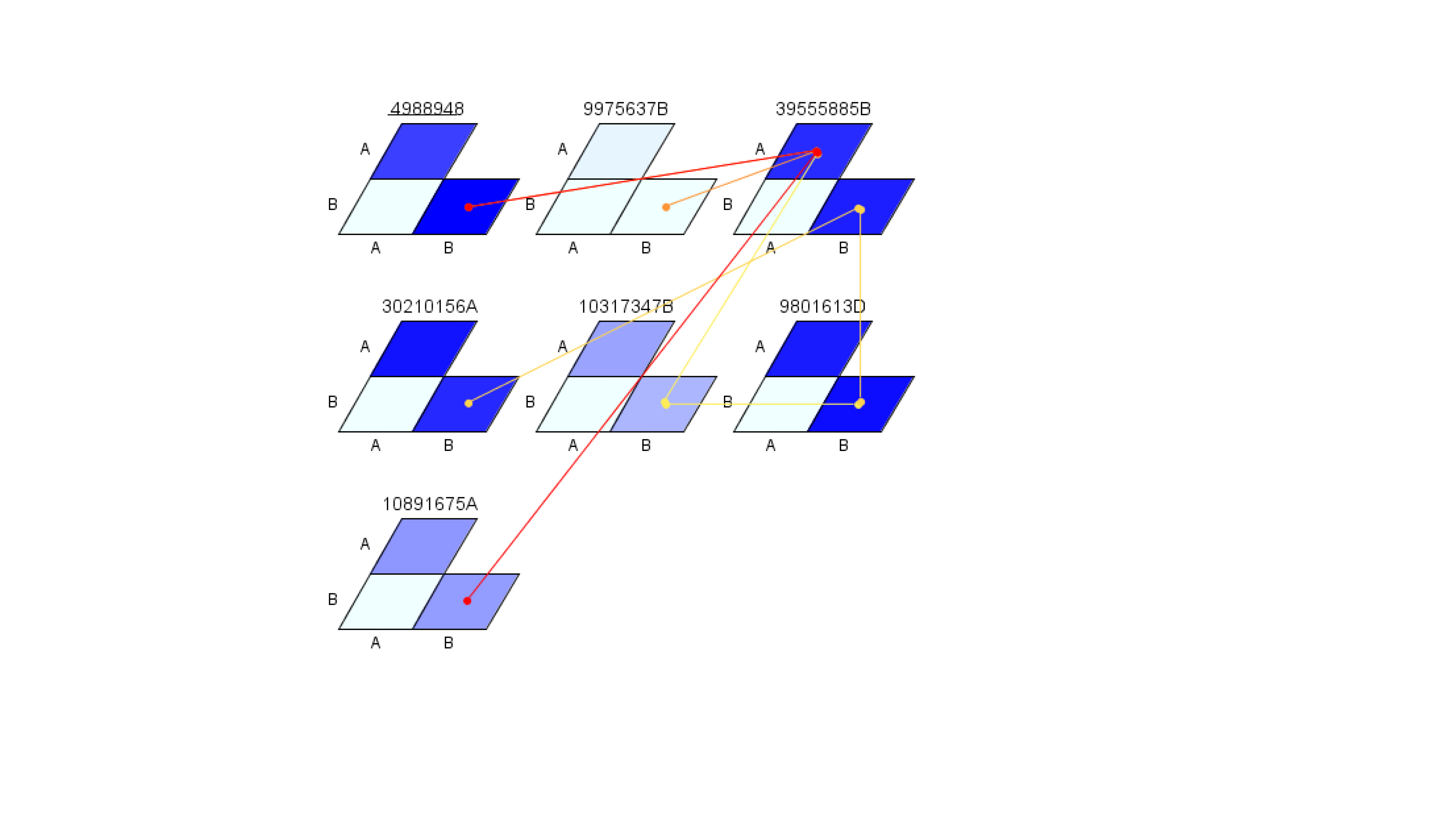

Supplement: Supplementary Figure 6 — Genome wide epistatic interactions for AUDPC 2018_infect; 1 associated SNP on 3A showed genome wide and explained 5.5%. [file Image_6.TIF]
