# Supplementary material for: Genome-Wide Association Study of Tan Spot Resistance in a Hexaploid Wheat Collection From Kazakhstan
Source: Front Genet. 2021 Jan 11;11:581214. doi: 10.3389/fgene.2020.581214 (PMC7831376; doi:10.3389/fgene.2020.581214)
Supplement: Supplementary Table 2 — Analysis of variance for tan spot (TS) disease scores for resistance to Race 1 and Race 5 and distribution of tan spot score in checks and the panel. [file Table_2.doc]

**Supplementary Table S2 |** Analysis of variance for tan spot (TS) disease scores for resistance to Race 1 and Race 5 and distribution

of tan spot score in checks and the panel

| Experiment | | Source | | Df | | Sum of square | | | Mean of square | | F value | | Ptr > F | Mean TS scores in checks  Glenlea Salamouni | | TS scores in the panel  Min Max Mean | | |
| --- | --- | --- | --- | --- | --- | --- | --- | --- | --- | --- | --- | --- | --- | --- | --- | --- | --- | --- |
| Race 1 | | Replication | | 7 | | 1.72 | | | 0.2460 | | 1.2987 | | 0.2295 | 4.5 | 1.1 | 1.0 | 4.9 | 2.9 |
|  |  | Genotype | | 190 | | 1957.22 | | | 10.3011 | | 54.3881 | | P<0.001 |  |  |  |  |  |
|  |  | Residuals | | 1330 | | 251.90 | | | 0.1894 | |  | |  |  |  |  |  |  |
|  | |  | |  | |  | | |  | |  | |  | 6B662 | Salamouni | 1.0 | 4.3 | 1.8 |
| Race 5 | | Replication | | 7 | | 2.24 | | | 0.32 | | 1.7968 | | 0.18409 | 4.3 | 1.1 |  |  |  |
|  |  | Genotype | | 190 | | 661.29 | | | 3.4805 | | 19.5414 | | P<0.001 |  |  |  |  |  |
|  |  | Residuals | | 1330 | | 236.88 | | | 0.1781 | |  | |  |  |  |  |  |  |
|  |  | |  | |  | |  |  | |  | |  | | | | | | |
|  |  | |  | |  | |  |  | |  | |  | | | | | | |
