# Supplementary material for: Genome-Wide Association Study of Tan Spot Resistance in a Hexaploid Wheat Collection From Kazakhstan
Source: Front Genet. 2021 Jan 11;11:581214. doi: 10.3389/fgene.2020.581214 (PMC7831376; doi:10.3389/fgene.2020.581214)
Supplement: Supplementary Table 3 — Analysis of variance (ANOVA) for tan spot severity and phenological traits under natural conditions and under artificial inoculation conditions and summary statistics. [file Table_3.doc]

**Supplementary Table S3.** Analysis of variance (ANOVA) for tan spot severity and phenological traits under natural conditions and under artificial inoculation conditions and summary statistics

| Experiment | Source | Df | Sum of square | Mean of square | F value | Ptr > F | Summary statistics | | | | Heritability |
| --- | --- | --- | --- | --- | --- | --- | --- | --- | --- | --- | --- |
|  |  |  |  |  |  |  | Min | Max | Mean | LSD |  |
| Tan spot - 2016 | Replication | 2 | 2233 | 1116.54 | 27.4909 | 0,7071 | 0 | 60 | 19,24 | 10.23 | 0.83 |
|  | Genotype | 190 | 48011 | 252.69 | 6.2216 | P<0.0000 |  |  |  |  |  |
|  | Residuals | 380 | 15434 | 40.61 |  |  |  |  |  |  |  |
| Tan spot - 2018 | Replication | 2 | 8079 | 4039,4 | 162,067 | 0,2201 | 0 | 75 | 19,03 | 8,01 | 0.91 |
|  | Genotype | 190 | 58987 | 310,5 | 12,456 | P<0.0000 |  |  |  |  |  |
|  | Residuals | 380 | 9471 | 24,9 |  |  |  |  |  |  |  |
| Tan spot -2018 inf | Replication | 2 | 5768 | 2884,08 | 36,8198 | 0,0242 | 0 | 90 | 26,77 | 14,21 | 0.87 |
|  | Genotype | 190 | 121094 | 637,34 | 8,1366 | P<0.0000 |  |  |  |  |  |
|  | Residuals | 380 | 29765 | 78,33 |  |  |  |  |  |  |  |
| Days to heading - 2016 | Replication | 2 | 370 | 184,76 | 1,6279 | 0,1977 | 221 | 338 | 233,37 | 10,14 | 0.77 |
|  | Genotype | 190 | 49326 | 259,61 | 2,2874 | P<0.0000 |  |  |  |  |  |
|  | Residuals | 380 | 43129 | 113,5 |  |  |  |  |  |  |  |
| Days to heading - 2018 | Replication | 2 | 257 | 128,504 | 5,057 | 0,0168 | 220 | 336 | 232,52 | 8,09 | 0.70 |
|  | Genotype | 190 | 16495,7 | 86,819 | 3,4166 | P<0.0000 |  |  |  |  |  |
|  | Residuals | 380 | 9656,3 | 25,411 |  |  |  |  |  |  |  |
| Days to heading - 2018Inf | Replication | 2 | 794 | 397,08 | 4,8704 | 0,0816 | 223 | 251 | 236,98 | 2,96 | 0.96 |
|  | Genotype | 190 | 33411 | 175,85 | 2,1568 | P<0.0000 |  |  |  |  |  |
|  | Residuals | 380 | 30981 | 81,53 |  |  |  |  |  |  |  |
| Plant height - 2016 | Replication | 2 | 1154 | 577,07 | 42,264 | 0,2217 | 50 | 124 | 84,82 | 5,93 | 0.98 |
|  | Genotype | 190 | 176229 | 927,52 | 67,93 | P<0.0000 |  |  |  |  |  |
|  | Residuals | 380 | 5189 | 13,65 |  |  |  |  |  |  |  |
| Plant height - 2018 | Replication | 2 | 520 | 259,89 | 19,326 | 0,1016 | 52 | 131 | 89,41 | 5,89 | 0.98 |
|  | Genotype | 190 | 176390 | 928,37 | 69,034 | P<0.0000 |  |  |  |  |  |
|  | Residuals | 380 | 5110 | 13,45 |  |  |  |  |  |  |  |
| Plant height - 2018 inf | Replication | 2 | 154 | 77,08 | 10,098 | 0,0534 | 51 | 133 | 91,15 | 4,43 | 0.98 |
|  | Genotype | 190 | 179014 | 942,18 | 123,436 | P<0.0000 |  |  |  |  |  |
|  | Residuals | 380 | 2901 | 7,63 |  |  |  |  |  |  |  |
