# Supplementary material for: Genome-Wide Association Study of Tan Spot Resistance in a Hexaploid Wheat Collection From Kazakhstan
Source: Front Genet. 2021 Jan 11;11:581214. doi: 10.3389/fgene.2020.581214 (PMC7831376; doi:10.3389/fgene.2020.581214)
Supplement: Supplementary Table 6 — Epistatic interactions among associated loci for tan spot resistance. [file Table_6.DOC]

**Table S6** Epistatic interactions among associated loci for tan spot resistance

| Trait | Interacting loci | R^2^ (two-loci) | R^2^ (three-loci) |
| --- | --- | --- | --- |
| Race 1 | 3021234, 1406319 | 0.10783 |  |
|  | 1406319, 1081730 | 0.11971 |  |
|  | 1406319, 3946488 | 0.15293 |  |
|  | 3021234, 1406319, 1081730 |  | 0.14169 |
|  | 3021234, 1406319, 3946488 |  | 0.18321 |
|  | 1406319, 1081730, 3946488 |  | 0.18773 |
| Race 5 | 1008802, 1862737 |  | 0.16034 |
|  | 1862737, 1093048 |  | 0.1685 |
|  | 1008802, 1862737, 1093048 |  | 0.21113 |
| Insensitivity to ToxA | - | - | - |
| Insensitivity to ToxB | - | - | - |
| PtrAUDP2016 | - | - | - |
| PtrAUDP2018 | - | - | - |
| PtrAUDP2018Infect | - | - | - |
